# Opportunities for digital health technology: identifying unmet needs for bipolar misdiagnosis and depression care management

**DOI:** 10.3389/fdgth.2023.1221754

**Published:** 2023-09-12

**Authors:** Sarah M. Kark, Michelle A. Worthington, Richard H. Christie, Aaron J. Masino

**Affiliations:** ^1^ AiCure, New York, NY, United States; ^2^ Department of Psychology, Yale University, New Haven, CT, United States; ^3^ Department of Biostatistics, Epidemiology, and Biomedical Informatics, Perelman School of Medicine, University of Pennsylvania, Philadelphia, PA, United States

**Keywords:** digital health technology, qualitative research, semi-structured interviews, psychiatry, needs assessment, bipolar misdiagnosis, digital tool development, text analysis

## Abstract

**Introduction:**

Digital health technologies (DHTs) driven by artificial intelligence applications, particularly those including predictive models derived with machine learning methods, have garnered substantial attention and financial investment in recent years. Yet, there is little evidence of widespread adoption and scant proof of gains in patient health outcomes. One factor of this paradox is the disconnect between DHT developers and digital health ecosystem stakeholders, which can result in developing technologies that are highly sophisticated but clinically irrelevant. Here, we aimed to uncover challenges faced by psychiatrists treating patients with major depressive disorder (MDD). Specifically, we focused on challenges psychiatrists raised about bipolar disorder (BD) misdiagnosis.

**Methods:**

We conducted semi-structured interviews with 10 United States–based psychiatrists. We applied text and thematic analysis to the resulting interview transcripts.

**Results:**

Three main themes emerged: (1) BD is often misdiagnosed, (2) information crucial to evaluating BD is often occluded from clinical observation, and (3) BD misdiagnosis has important treatment implications.

**Discussion:**

Using upstream stakeholder engagement methods, we were able to identify a narrow, unforeseen, and clinically relevant problem. We propose an organizing framework for development of digital tools based upon clinician-identified unmet need.

## 1. Introduction

Digital health technologies (DHTs) driven by artificial intelligence (AI) applications hold the potential to transform healthcare efficiency and substantially improve patient health outcomes at scale. The hope that DHTs will lead to a new era of effective and high-value patient care is reflected in recent industry funding and resource investments. Most major digital companies have announced AI-driven healthcare initiatives (1), and 29.1 billion dollars were invested in digital health start-ups in 2021—more than three times the pre-COVID-19 pandemic levels of 8.2 billion dollars in 2019 (2). However, despite billions of dollars invested and major technological advances, there is paradoxically very little evidence of widespread clinical uptake or proof of broad-scale gains in patient health outcomes (3, 4). To date, DHTs have largely fallen short on their potential, suggesting a disconnect between the envisioned clinical impact and real-world value (4, 5). This disconnect will not likely be bridged by further improving the accuracy of AI approaches themselves, but rather by careful consideration of addressing real-world needs to promote positive behavioral changes (1).

The apparent disconnect between resource allocation and real-world clinical impact is in part related to a lack of interaction between and misalignment of priorities among stakeholders within the digital health ecosystem (e.g., patients, providers, digital health companies, payers, investors) (4). Most DHT developers are working at large technology companies, start-ups, or in academic research departments, developing concepts and prototypes without a firsthand understanding of the clinical experience of clinicians or patients (3). That is, shortcomings in AI-driven healthcare can occur when ideas are conceived of in isolation from key digital ecosystem stakeholders and then retrofitted to a perceived clinical problem based on uninformed assumptions, leaving real-world problems unaddressed and possibly entirely unidentified in the first place. No matter how accurately the AI-enabled system performs, or how beautified the data delivery display, the clinical impact will fall short if it fails to address an actual clinician-defined need. In recognition of this issue, recent work demonstrates utilizing methods of upstream stakeholder engagement, such as qualitative research studies, to identity and drive the design of AI-based tools to increase their clinical relevance as well as potential for widespread uptake into clinical practice (3, 6, 7). Qualitative research methods enable new knowledge creation through serendipitous discovery of unforeseen challenges, testing and reconceptualization of prior knowledge, and the ability to formulate valuable questions (8). There is already some work applying qualitative research for digital tools in depression (9, 10). However, to our knowledge, many studies assess provider and patient perceptions of *existing* hypothetical tools, rather than using qualitative research upstream to generate foundational ideas from which to conceptualize and build new DHTs.

We recently demonstrated the utility of applying upstream qualitative interview methods to uncover challenges that psychiatrists face when treating outpatients with major depressive disorder (MDD) (11, *ISCTM*). MDD is one of the most common neuropsychiatric disorders and is largely characterized by depressed mood and/or the reduced ability to experience pleasure (i.e., anhedonia) for at least two consecutive weeks. Beyond these cardinal symptoms, there is vast symptom heterogeneity in MDD (12), with five required symptom types out of nine possible categories (e.g., sleep, appetite, psychomotor changes) for diagnosis—leading to 1,000 unique combinations (13). The non-specificity of MDD poses a challenge to effective care management. Large numbers of research publications listed on PubMed are dedicated to subtyping MDD. Since the year 2000, over 3,000 articles have been published that include “subtype OR subtyping” and “depression OR MDD” in the title or abstract (PubMed.gov, accessed 16 August 2023). Over the past decade, a paper has been published nearly every other day with regard to MDD subtyping research. Based on our own extensive literature review, we originally hypothesized that an AI-driven patient-facing DHT designed to assist psychiatrists in subtyping their MDD patients would meet a real-world challenge. We interviewed psychiatrists to both test this hypothesis and ask a series of open-ended questions to uncover unanticipated challenges. Surprisingly, we found that precision subtyping of MDD was not a necessity as perceived by providers. However, using text analysis of the interview transcripts and thematic analysis, we uncovered frequent utterances related to the challenges associated with medication side effects, treatment resistance, comorbid substance use, and bipolar disorder (BD). Discussing each of these topics in-depth was outside the scope of our prior work. However, without further systematic analysis of the specific content related to BD, we could not decipher why and how BD posed a challenge to the clinical management of MDD. Here, we focus on identifying and defining the context around interviewee utterances related to BD.

BD and other related disorders are well characterized in the literature (14, 15). While MDD patients experience depressive episodes without manic symptoms, BD is characterized by severe, persistent, and recurring alternations between periods of manic and depressive mood states. BD I is marked by manic episodes lasting a week or more that are characterized by abnormal levels of elation, extreme mood states, elevated energy with a decreased need for sleep, extreme confidence, self-grandiosity, and talkativeness that often involve psychotic elements (16). During a manic episode, patients tend to experience severely impaired judgement and reduced impulse control, which can sometimes result to hospitalization or negative interactions with law enforcement (17). By contrast, BD II is characterized by depressive episodes with alternative hypomanic episodes. Hypomanic episodes are less intense than full manic episodes, do not exhibit psychotic elements, and tend to be shorter than manic episodes (e.g., four consecutive days). During a hypomanic episode, patients can find the elevated mood state and reduced need for sleep to be positive and even productive, also known as positive-productive symptoms (18). However, there are also problem-causing symptoms of hypomania, such as increased risk-taking, substance use, irritability, and excessive shopping and spending (19). Together, BD poses a major economic, societal, and humanistic burden (20, 21).

In the present study, we sought to identify the challenges in managing MDD care for digital tool design; specifically, we asked: Why did most of the psychiatrists spontaneously refer to BD in an interview focused on managing MDD care? To systematically identify and define the importance of BD, we applied text analysis to extract context from the sentences immediately surrounding all utterances of “bipolar” and related keywords (“mania,” “manic”) and subjected those text segments to qualitative thematic analysis. The aim of this work was threefold: (1) demonstrate the utility of methods of upstream stakeholder engagement to elucidate the unforeseen challenges and unmet needs that clinicians face in real-world clinical practice, (2) utilize the resulting themes to identify and define the importance of BD in managing MDD care, and (3) propose an organizing framework for early-stage design of digital tools based on clinician-identified unmet clinical needs.

## 2. Materials and methods

### 2.1. Ethics statement

The present study is a retrospective analysis of interview data originally collected for market research purposes. The BRANY Institutional Review Board (IRB) determined that this study is exempt from the IRB review under category 4ii in 45 CFR 46.104(d) as secondary research for which a written consent is not required.

### 2.2. Sample and design

The interviews were conducted with 10 psychiatrists originally recruited for market research purposes using the Guidepoint Expert Network (Guidepoint Global, LLC, New York, NY, USA). We interviewed 10 experienced psychiatrists treating 20 or more MDD patients per week in the United States. With only two exceptions, we interviewed psychiatrists with 10 or more years of experience post-residency who also spent most of their time (50%+) on direct outpatient patient care. Based on prior work, we estimated that 9–17 interviews (22) would be required to reach theoretical saturation (23)—the point during data collection in which additional insights wane, data begin to repeat, and further data collection becomes redundant. Indeed, after 10 interviews, we observed repeating themes that appeared in 50%–100% of the interviews, including medication side effects, substance use, energy levels, treatment resistance, BD, and family history (11, *ISCTM*). Moreover, our inclusion criteria targeted a relatively homogenous sample of psychiatrists, and we did not require sample sizes to generate any between-subject comparisons.

Based on the aims of the research, the first author wrote the initial set of interview questions and possible probes for the interview guide, which was subsequently reviewed, revised, and approved by the study team. The first author used the interview guide (see Box 1 for sample questions and Supplementary Materials for the full interview guide) to conduct 1-h, semi-structured, audio interviews using Zoom. Interview transcript text files were automatically generated by Zoom and subsequently imported into MAXQDA Analytics Pro 2022 Release 22.1.1 for manual quality checking and further analysis.

BOX 1 **Sample semi-structured interview questions.**

• Can you describe your typical care management process for an MDD patient?
• What are the most important pieces of information you gather about a patient?
• What are the key pieces of information for diagnosis?
• How do you gather this information from your patients?
• Can you tell me about how to differentiate between depressive symptoms and other underlying health problems, comorbid medical issues?
• Can you tell me about how to differentiate between depressive symptoms and other psychiatric comorbidities?
• Can you please describe a few specific cases? What kinds of medications did you try with them over time and why? How long did it take to find a resolution?

### 2.3. Text analysis

Based on our prior work [ISCTM (11)] showing that half of the psychiatrists uttered the bigram “bipolar depression” at least once during their interview, here we conducted qualitative analysis of the text immediately surrounding utterances of “bipolar,” “manic,” and “mania”. We used MAXQDA to pull keyword use and the 15 words preceding and following each utterance. These text segments were exported for further review and analysis by the two researchers who were familiar with the interviews (one researcher had conducted the interviews firsthand, and the second researcher had listened to and read the transcripts several times). Occasionally, the whole paragraph including an utterance was referenced to provide more context.

### 2.4. Theme and sub-theme identification

The primary goal of this research was to identify unforeseen emergent themes, as opposed to applying a priori theory. Thus, our approach was inspired by inductive analysis—a “bottom-up” strategy for theme identification—guided by grounded theory (23). Inductive analysis involves iteratively reading and re-reading interview transcripts to generate and continuously refine themes and sub-themes (or codes) that progressively provide a structure to the raw data (24, 25). Here, the emergent themes and sub-themes then entered a loop-like deductive–inductive analysis pattern over several rounds of discussion and revision until the authors reached full agreement on the theme and sub-theme assignments (26).

Specifically, analysis began with one researcher applying inductive analysis to the outputted text segments to identify, name, and define themes and sub-themes based on the content of the outputted text segments. Second, a second researcher independently used the sub-themes definition table generated by the first researcher to deductively label each outputted text segment with the best-fitting sub-theme based on their definitions. Third, inter-rater agreement between the first and second researcher was calculated using the unweighted Cohen's kappa statistic for nominal data implemented in JASP version 0.16.3 (University of Amsterdam). Fourth, the full research team met to further define and agree upon the sub-themes and their definitions and make adjustments as necessary. At this stage in the cycle, overarching themes were inductively identified in order to nest the sub-themes. Fifth, the two original researchers independently re-scored the text segments based on the updated definition table, and inter-rater agreement was calculated again. Lastly, the first and second author met iteratively to resolve any discordant text segment sub-theme assignments and refine the sub-themes definition table as necessary until perfect agreement was achieved.

## 3. Results

### 3.1. Sample

We interviewed 10 English-speaking psychiatrists recruited from three distinct treatment settings from across the United States (Group/Private Practice: *n* = 4; Academic Medical Center/University Teaching Hospital: *n* = 3; and Community Hospitals: *n* = 3) with a range of years of experience post-residency, weekly outpatient load, and direct time allocation to MDD outpatients (see Table 1 for psychiatrist characteristics).

**Table 1:** Individual psychiatrist characteristics (*N* = 10).

Setting	Region	Clinic experience (years)	MDD patients/ week (count)	Direct patient care time (%)	Outpatient time (%)
Academic Medical Center/University Teaching Hospital	West	25	250	90	80
	Northeast	25	120	75	75
	Southwest	20	20	30	99
Community Hospital	Midwest	25	35	95	50
	Southwest	10	35	98	60
	Southwest	7	50	100	50
Group/Private Practice	West	28	35	95	95
	Mid-Atlantic	13	50	90	85
	Southwest	13	40	95	100
	West	12	30	100	100

### 3.2. Text analysis

The text analysis revealed 67 instances of the keywords (“bipolar,” “manic,” “mania”) across eight documents, demonstrating that eight out of ten advisors uttered one of the three keywords at least once. We initially identified and removed two instances in which the use of a keyword was used in a clarifying question, resulting in 65 keyword utterances and their surrounding context for further consideration in the qualitative analysis.

### 3.3. Theme and sub-theme identification

After reviewing the 65 resulting text segments, the first researcher (SK) identified 12 sub-themes describing the content and meaning of the text segments. Next, the sub-theme definitions were given to the second researcher (MW) who used the sub-theme definitions to label each of the 65 text segments to a defined sub-theme. After this initial rating stage, the inter-rater agreement was “substantial” [unweighted Cohen's kappa statistic = 0.74, SE = 0.06, 95% CIs (0.63–0.86)]. The two researchers agreed on 51 out of 65 segments (78.5%).

Next, the full research team reviewed the sub-theme table, and reconceptualized the 12 sub-themes down to eight sub-themes nested under three main overarching themes: Diagnosis, Barriers to Information Acquisition, and Treatment Implications (see Table 2). At this stage, three more text segments were identified and excluded from further analysis (one instance of a keyword embedded in a clarifying statement and two instances of an advisor referring to their training background), reducing the final number of text segments for further analysis to 62.

**Table 2:** Themes and sub-themes identified from the thematic analysis of text segments surrounding the 62 keyword utterances, example quotes, and sub-theme frequencies and usage across psychiatrists.

Theme	Sub-theme	Description	Utterances		Psychiatrists	
			Count	%	Count	%
Diagnosis	Key information to confidence in MDD diagnosis	BD symptom insight is a crucial piece of information to feel confident in unipolar MDD diagnosis	11	17.7	5	50
	Reference to misdiagnosis as a challenge	Specific acknowledgment of the BD misdiagnosis challenge (e.g., inability to distinguish MDD from BD patients during depressive episodes or contribution to MDD treatment resistance)	9	14.5	5	50
	Specific BD symptoms or history	Examples of symptoms or history that would implicate possible BD	22	35.5	4	40
	General evaluation and screening	General discussion of screening (e.g., DSM-5 criteria, along with other disorders or comorbidities)	3	4.8	3	30
Barriers to information acquisition	Lack of clinical observation	Reference to patients who do not come to the clinic when manic	6	9.7	3	30
	Patient self-report	Patients’ ability or self-insight to report symptoms related to mania	2	3.2	2	20
Treatment	Implications for treatment	Reference to differential treatment for unipolar compared with BD patients, medications, or antidepressant-induced mania	8	12.9	6	60
Other	Patient concerns	Patient worried about becoming manic	1	1.6	1	10

The utterances count refers to the number segments assigned to a particular sub-theme. Psychiatrists count refers to the number of psychiatrists (out of 10) who had one or more segments assigned to a particular sub-theme (e.g., two out of 10 advisors had segments labeled with the “Patient self-report” sub-theme).

Finally, the two researchers who initially coded the segments used the final sub-theme definition table to independently re-code the text segments without referencing the original coding. After this second rating stage, the rater agreement between the two authors was “strong” [unweighted Cohen's kappa statistic = 0.82, SE = 0.05, 95% CIs (0.71–0.92)]. The two researchers agreed on 53 out of 62 segments (85.5%), suggesting robustness and clarity of the final sub-theme definitions. Finally, the two researchers met to discuss the nine instances of discordance until they achieved perfect agreement. The final themes, sub-themes, definitions, utterance frequencies, and psychiatrist use of themes and sub-themes are summarized in Table 2.

We found that the majority of keyword uses [73% (45 utterances)] centered around issues of MDD diagnosis and bipolar misdiagnosis, including (1) emphasis on how knowledge of any bipolar symptoms is critical to making a diagnosis of unipolar MDD, (2) direct reference to bipolar misdiagnosis as a challenge in psychiatry, (3) detailed explanations of specific symptoms and behaviors in BD, and (4) general and brief references to screening for BD. For the purposes of this work, we focus on the finding that the ability to “rule out” bipolar disorder was one of the most important pieces of information when making an accurate MDD diagnosis, and being able to do so increased the psychiatrists’ confidence in their MDD diagnosis. Half of the psychiatrists interviewed in this study stated not only how important it is for a confident MDD diagnosis to “make sure” there was no evidence of BD, but also how difficult it is to “rule out” bipolar in patients presenting with primary complaints of depression. For example, one psychiatrist stated:

“*Definitely a big one will be to rule out bipolar disorder or mania. That's the big one, I need to make sure I don’t miss.*”

“*But by and large … [for] the diagnosis of unipolar depression [we] certainly want to rule out bipolar depression as a main key piece.*”

In a direct response to the interview question, “What kind of information makes you feel confident in how you choose a first medication?” one psychiatrist stated:

“*[You’re] never 100% confident, but if really there's no suggestive evidence of bipolarity as best as you can tell and the history's suggestive of a major depressive episode, then you can at least think that it's a reasonable first choice to go with an antidepressant.*”

Further, the psychiatrists warned that patients are commonly misdiagnosed with unipolar depression and that treatment-resistant MDD can be linked to undetected—and therefore untreated—bipolar disorder in some cases. Yet, in the depressed mood phase, MDD and BD are indistinguishable. For example:

“*You want to make sure it's not a bipolar spectrum condition … a lot of doctors might diagnose them as not a bipolar type, but you know, just unipolar depression.*”

“*… sometimes when people do come in and they do have treatment-resistant depression, there might be a little bit of a subtle bipolar and that's why things aren’t working.*”

“*When they’re in the throes of depression, the bipolar depression and the MDD depression, they look they look the same. I don’t think I can really tell.*”

The second theme based on text analysis of three advisors occurred less frequently overall, but it importantly highlights two types of barriers to providers accessing critical information that is key for accurate clinical evaluation. We found that clinical information is occluded from psychiatrist observation for at least two reasons: First, bipolar patients tend to appear to the clinic when they are in the depressive state, not when they are experiencing symptoms of mania or hypomania, removing the opportunity for physical or well-timed telehealth observation of manic or hypomanic symptoms. For example, one advisor stated:

“*We usually see them in the depressed or the mixed episodes. Not so much the manic episode. Certainly not so much the hypomanic. I mean, if they’re full blown manic they may end up in the ER.*”

The other reason is that some patients do not report symptoms related to mania because they lack insight that these behaviors are even clinically relevant. For instance,

“*First of all, no bipolar [patient] comes in when they’re manic and says I’ve got a lot of energy, I’m very important, and I’m doing things really well, they just don’t see it. So, mania is really not in their lexicon to come in … and tell you ‘[I’m] needing stabilization’.*”

Thus, psychiatrists often rely on self-report of symptoms and behaviors that are typically not observed directly in the clinic.

Although there were only eight total utterances related to treatment implications, this third theme held the most consensus across the psychiatrists (60%). Specifically, these utterances highlighted how patients with BD and MDD need to be treated and monitored differently and that there is a risk of treatment-emergent mania or hypomania with antidepressant (AD) monotherapy in undiagnosed BD patients. For example:

“*If somebody becomes depressed and they’ve had manic episodes you treat them very differently than somebody that hasn’t had any manic episodes.*”

“*If they’re experiencing just really too many side effects, if their mood is getting worse, if they’re becoming suicidal, they’re becoming sort of hypomanic or things like that, those would indicate it may be time to change.*”

Finally, another psychiatrist elaborated on behaviors related to treatment-emergent mania that also highlighted the issues of patient self-report:

“*Let's say I gave them [an anti-depressant medication] and then they went home, and they got manic, and they loved it. So, they’re not telling me that they’re not sleeping they’re spending their money, they’re gambling in the casino, they’re telling everybody that they’re rich, rich, rich. And they’re having promiscuous sex and they come in, they go: ‘This pill is the greatest thing that ever happened for me it's working. I’m doing well, I don’t need to take anything else, it's perfect’.*”

## 4. Discussion

**Figure 1:**
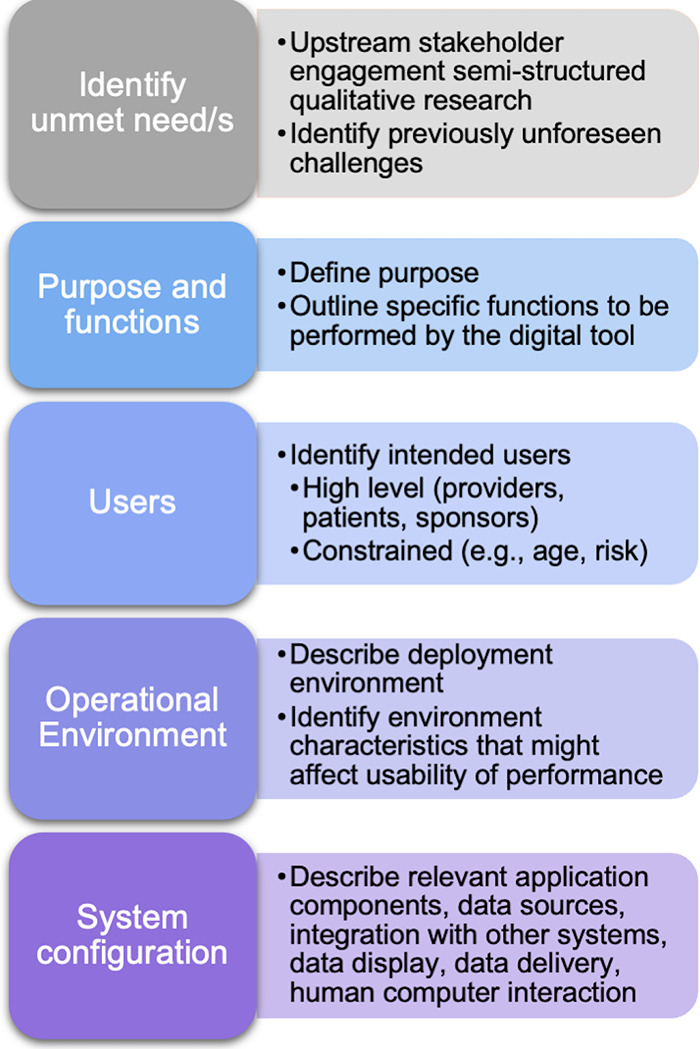
Proposed organizing framework for developing digital tools based upon a clinician-identified unmet need.

The current qualitative research study used methods of upstream stakeholder engagement to uncover a very specific, real-world challenge: BD misdiagnosis in patients presenting with MDD symptoms. Most psychiatrists in this study (80%) spontaneously elaborated on issues related to BD misdiagnosis. Specifically, we found that a correct BD diagnosis is both highly important but also very difficult due to barriers in information acquisition. This combination of importance and difficulty flags this issue as an unmet need and a worthy problem for DHT development. Next, we use these findings as a case example to demonstrate an early-stage organizing framework for developing digital tools based upon a clinician-identified unmet need (see Figure 1).

The current methods enabled the first necessary step for creating a clinically impactful digital tool: identification of an ongoing, real-world challenge with a sufficiently narrow scope. The unmet needs identified here are echoed in contemporary psychiatry literature, which demonstrates the strength of this method in guiding DHT developer awareness to existing challenges. However, despite an extensive literature review prior to this study, we were unaware of this particular area of psychiatry literature. A follow-up literature review guided by the current findings showed that the rate of undiagnosed bipolar in MDD patients (i.e., BD misdiagnosis) is 17%–50% (27, 28). Most misdiagnosed bipolar patients initially present with depression symptoms and receive an MDD diagnosis, which often results in treating with AD monotherapy without mood stabilizers (29, 30), which is sub-optimal and also raises the risk for treatment-emergent mania (28, 29, 31). Timely BD diagnosis is crucial because even milder forms of unmanaged BD can progress to become more severe (32). However, there is an alarmingly common protracted time of 5–10 years between onset of BD and clinical management (30, 33–35). Patients typically see four physicians and receive three to four incorrect diagnoses before they receive a correct BD diagnosis (30, 36). Delayed diagnosis is associated with an increased personal, humanistic, and socioeconomic burden, including increases in suicidality, social and cognitive functional impairments, and hospitalizations (29, 30, 36, 37). Thus, there is an urgent need to reduce time to accurate BD diagnosis.

Despite a vast literature and evidence-based risk-factors and features that can aid in distinguishing BD from unipolar MDD, it is evident from the present study that BD misdiagnosis challenges persist in contemporary clinical practice. The psychiatrists in this study highlighted some of these features, such as a positive family history of BD, early age of onset, depression severity, treatment resistance, sleep patterns, and legal or financial problems. These are known risk factors consistent with the literature along with other factors with diagnostic utility such as atypical depressive features (e.g., hypersomnia), psychomotor retardation, and substance use (29, 30, 38, 39).

Although there are known risk-factors, practicing psychiatrists have difficulty accessing the key information needed to confirm them and make a BD diagnosis. The psychiatrists in the current study illuminated at least two factors that contribute to this challenge: (1) lack of clinical observation and (2) reliance on patient self-report. Both factors occlude critical information from clinical view. Self-report places a burden on patients to be an accurate historian of their own symptoms (37), despite known memory impairments in BD (40). Moreover, patients might not recognize their manic symptoms as clinically relevant to their primary complaints of depression (i.e., “not in their lexicon”), and others might intentionally conceal manic or hypomanic symptoms due to embarrassment, denial of illness, social self-stigma, or even the positive-productive aspects of hypomania (37, 41). Together, these findings point toward a need to connect physicians with patients for timely clinical observation based on objective measures that do not rely solely on subjective self-report. Next, we walk through initial steps in organizing key information for developing digital tools (Figure 1).

After identifying a problem, the next step is to clearly define the purpose and function/s to be performed by the DHT. What will the technology do to address the unmet need? In this case, one potential purpose to meet this challenge would be to minimize the delay to diagnosis in misdiagnosed bipolar patients. An example function could be to use smartphone-based digital traits to detect hypomanic and manic mood epochs during pre-visit and treatment periods. This function could serve to connect the patient and provider for evaluation during otherwise unobserved mood change epochs—bringing the symptoms into view for the clinician, enabling shorter duration to diagnosis and treatment of BD. Broadly, the tool could leverage objective, naturalistic, longitudinal, experience sampling data collected from the patient to connect physicians and patients for a timely observation by notifying the provider or their staff to contact the patient for a telehealth or in-clinic visit. Providers could potentially also opt to review key data sources or inference model features that drove a particular notification.

The next step is to carefully consider the patient and provider user population based on the purpose and function/s. Who are the intended users? Here, we interviewed psychiatrists from various settings managing adult patients with primary complaints of depression. One might constrain their patient population based on demographic factors such as age (e.g., adolescent, adult, older adults) or by the presence or absence of risk factors for the disease of interest (e.g., family history, genetic profile). For feasibility, one might consider a layered deployment approach—first validating the tool in a more constrained population or specific setting (e.g., MDD patients seen in an academic hospital with a positive family history of BD) to first test the false positive rates and characterize its sensitivity and specificity before expanding to broader use cases (e.g., patients with depressive symptoms presenting to primary care settings with no family of BD).

Next, digital tools operate within physical environments that require consideration at the outset. Where will the tool operate? In the case of a BD misdiagnosis tool based on experience sampling meant to capture out-of-clinic behavior and functioning, it should operate within the patient's natural setting (e.g., work, home, school) on a typical mobile device, and the provider portal will operate in the healthcare office setting. Further, it is crucial to anticipate the characteristics of the environment that might impact usability or performance. For example, if a function of the tool is to engage patients in a simulated conversation to detect changes in speech patterns, this would require text-to-speech conversion models to operate on the patient's device. For the physician, the results and notifications will need to integrate seamlessly into their workflow to avoid burden—a major barrier to clinical utility uptake emphasized by psychiatrists in this study and elsewhere (4).

The last stage of this framework is to identify and describe the system configuration, including relevant application components, data sources, integrating with other systems, data display, data delivery, and human computer interaction. For instance, including a simulated dynamic conversation feature would require a conversational chat bot component to engage patients on a daily basis, backend analytical processing to detect signs of manic or hypomanic behavior based on their responses, and integration into the electronic medical record (EMR) for seamless data reporting to the physician. Regarding data sources, there are now myriad methods for collecting digital measures of health and functioning, such as passive and active data streams collected from smartphones, wearables, and sensors. Meaningful data sources will depend on the disease state as well as the purpose, functions, specific users, and operational environment. For BD misdiagnosis, one could consider focusing on BD risk factors and well-known BD prodromes, such as changes in energy levels, sleep patterns, and social functioning (29, 42, 43). Data sources for BD could draw on passively collected sleep tracking, cell phone usage, location data, episodic audio collection (44), physiological changes (45), personal financial behaviors (e.g., overspending, compulsive buying) (46, 47), vocal acoustics and speech characteristics (48–51), and ecological momentary assessments (EMA) for subjective experience ratings of emotion and functioning (52).

Passive patient data collection is unobtrusive and involves very little patient effort. While passive data collection yields high levels of adherence, disadvantages include privacy concerns (53), and data that are difficult to interpret without context provided by patients (54). For example, regarding the latter, a potential BD patient could show increased location movement and reduced phone usage for a few days because they are on a family road trip (i.e., not consistent with manic behavior) and not because they have intentionally turned off their phone to covertly drive long distances to start potential businesses (i.e., a behavior consistent with mania). By contrast, active tasks involve patient participation in a task (e.g., describe a picture, talk about their day, recall a memory), which can result in relatively lower adherence rates. However, active tasks can better elicit and capture variability associated with disease-relevant behaviors (55). To reduce burden, tasks can be designed to be lightweight and strategically scheduled. For example, effective assessment scheduling for BD could leverage known sleep–wake rhythmicity (32, 56–59) and apply an adaptive sampling algorithm to up-sample assessment frequency during suspected periods of mood change. Passive and active monitoring each have their respective trade-offs—the optimal solution is likely juxtaposition of the two data streams (60). In any case, these high-frequency data streams yield temporally dense data sets with a vast number of features, which invoke the need for specific analytical considerations, such as dimension reduction techniques and statistical modeling approaches that account for within-person autocorrelation [e.g., general linear mixed models (GLMMs) or generalized estimating equations (GEEs)] (61).

We recommend early anticipation and planning around issues of feasibility and privacy. For instance, 79% of BD patients are open to using apps for self-monitoring of symptoms (62), with some evidence that daily smartphone-based EMA is feasible for up to 2 years in some BD populations (63). BD patients describe privacy concerns as a top consideration for DHT use and may benefit from features such as privacy customization and insurances around tracking and targeted advertisements (64, 65). Specific considerations related to patient data and privacy should also guide digital tool development and implementation. Moreover, the specific tool functionality (e.g., intervention, diagnostic, communication) has implications for the legislation concerning patient data, patient safety, patient privacy, deliverance of healthcare, and regulation of medical devices (66).

Finally, it is important to acknowledge the limitations of the present study. First, although a sample size of 10 is within acceptable ranges (9–17 interviews) for reaching theoretical saturation in qualitative research (22), the data derived from 10 psychiatrists might not be sufficient to reveal certain unmet needs that were not identified here. Further, as we sampled from experienced psychiatrists from a range of settings, further work is needed to fully uncover and appreciate unmet needs that might differ as a function of clinical setting and clinician characteristics.

In conclusion, the present study demonstrates the importance of upstream engagement of stakeholders through qualitative research to reveal unforeseen and meaningful real-world challenges amenable to DHT solutions. Here, we outlined a framework for organizing initial design of digital tools to address unmet needs. The next steps would be to address these areas in detail and validate the tool. Future work is needed to engage patients, such that the digital solutions meet the overlapping needs of both patients and providers, which could maximize real-world clinical utility uptake and patient outcome impacts and bolster patient–clinician interactions and shared decision making.

## Data Availability

This article includes selected quotes from providers in the results table. The expanded dataset of de-identified text segments is available upon reasonable request. Requests to access these data should be directed to rich.christie@aicure.com

## References

[1] Emanuel EJ Wachter RM. Artificial intelligence in health care: will the value match the hype? JAMA. (2019) 321(23):2281–2. 10.1001/jama.2019.4914 31107500

[2] Krasniansky A Evans B Zweig M. 2021 year-end digital health funding: seismic shifts beneath the surface (2022). Available at: https://rockhealth.com/insights/2021-year-end-digital-health-funding-seismic-shifts-beneath-the-surface/ (Accessed October 18, 2022).

[3] Rudin RS Bates DW MacRae C. Accelerating innovation in health IT. N Engl J Med. (2016) 375(9):815–7. 10.1056/nejmp1606884 27579633 PMC6541913

[4] Lyles CR Adler-Milstein J Thao C Lisker S Nouri S Sarkar U. Alignment of key stakeholders’ priorities for patient-facing tools in digital health: mixed methods study. J Med Internet Res. (2021) 23:e24890. 10.2196/24890 34435966 PMC8430871

[5] Li RC Asch SM Shah NH. Developing a delivery science for artificial intelligence in healthcare. NPJ Digit Med. (2020) 3:107. 10.1038/s41746-020-00318-y 32885053 PMC7443141

[6] Corner A Pidgeon N Parkhill K. Perceptions of geoengineering: public attitudes, stakeholder perspectives, and the challenge of “upstream” engagement. Wiley Interdiscip Rev Clim Change. (2012) 3:451–66. 10.1002/wcc.176

[7] Tonekaboni S Joshi S Mccradden MD Goldenberg A Ai AG. What clinicians want: contextualizing explainable machine learning for clinical end use. In: Machine learning for healthcare conference. PMLR (2019). p. 359–80.

[8] Eakin JM Gladstone B. “Value-adding” analysis: doing more with qualitative data. Int J Qual Methods. (2020) 19:1609406920949333. 10.1177/1609406920949333

[9] Patoz MC Hidalgo-Mazzei D Blanc O Verdolini N Pacchiarotti I Murru A Patient and physician perspectives of a smartphone application for depression: a qualitative study. BMC Psychiatry. (2021) 21(1):65. 10.1186/s12888-021-03064-x 33514333 PMC7847000

[10] Silfee V Williams K Leber B Kogan J Nikolajski C Szigethy E Health care provider perspectives on the use of a digital behavioral health app to support patients: qualitative study. JMIR Form Res. (2021) 5(9):e28538. 10.2196/28538 34529583 PMC8512194

[11] Kark SM Worthington MA Molière K Epifano J Paredes AD Caamano S Insights into care management of major depressive disorder: upstream engagement methods identify unmet needs in psychiatry [poster abstract]. 2022 International society for CNS clinical trials and methodology autumn meeting; 2022, Sep 08-09. Boston, MA: The International Society for CNS Clinical Trials and Methodology (ISCTM) (2022).

[12] Harald B Gordon P. Meta-review of depressive subtyping models. J Affect Disord. (2012) 139:126–40. 10.1016/j.jad.2011.07.015 21885128

[13] Fried EI Nesse RM. Depression sum-scores don’t add up: why analyzing specific depression symptoms is essential. BMC Med. (2015) 13:72. 10.1186/s12916-015-0325-4 25879936 PMC4386095

[14] Carvalho AF Firth J Vieta E. Bipolar disorder. N Engl J Med. (2020) 383:58–66. 10.1056/NEJMra1906193 32609982

[15] NIMH. Bipolar disorder. National Institute of Mental Health (NIMH) (2022). Available at: https://www.nimh.nih.gov/health/topics/bipolar-disorder (Accessed October 18, 2022).

[16] Grande I Berk M Birmaher B Vieta E. Bipolar disorder. Lancet. (2016) 387:1561–72. 10.1016/S0140-6736(15)00241-X 26388529

[17] Connolly KR Thase ME. The clinical management of bipolar disorder: a review of evidence-based guidelines. Prim Care Companion J Clin Psychiatry. (2011) 13(4):PCC.10r01097. 10.4088/PCC.10r01097 PMC3219517 22132354

[18] Akiskal HS Hantouche EG Allilaire JF. Bipolar II with and without cyclothymic temperament: “dark” and “sunny” expressions of soft bipolarity. J Affect Disord. (2003) 73(1-2):49–57. 10.1016/S0165-0327(02)00320-8 12507737

[19] Hantouche EG Angst J Akiskal HS. Factor structure of hypomania: interrelationships with cyclothymia and the soft bipolar spectrum (2003). Available at: www.elsevier.com/locate/jad (Accessed October 20, 2022). 10.1016/s0165-0327(02)00319-1 12507736

[20] Jin H McCrone P. Cost-of-illness studies for bipolar disorder: systematic review of international studies. Pharmacoeconomics. (2015) 33(4):341–53. 10.1007/s40273-014-0250-y 25576148

[21] Sylvia LG Montana RE Deckersbach T Thase ME Tohen M Reilly-Harrington N Poor quality of life and functioning in bipolar disorder. Int J Bipolar Disord. (2017) 5(1):10. 10.1186/s40345-017-0078-4 28188565 PMC5366290

[22] Hennink M Kaiser BN. Sample sizes for saturation in qualitative research: a systematic review of empirical tests. Soc Sci Med. (2022) 292:114523. 10.1016/j.socscimed.2021.114523 34785096

[23] Glaser B Strauss A. The discovery of grounded theory: strategies for qualitative research. New Brunswick, NJ, USA: Aldine de Gruyter (1967).

[24] Shaw E. A guide to the qualitative research process: evidence from a small firm study. Qual Mark Res. (1999) 2(2):59–70. 10.1108/13522759910269973

[25] Srivastava P Hopwood N. A practical iterative framework for qualitative data analysis. Int J Qual Methods. (2009) 8(1):76–84. 10.1177/160940690900800107

[26] Berkowitz S. Analyzing qualitative data. In: Frechtling J Sharp L, editors. User-friendly handbook for mixed method evaluations. Division of research, evaluation and communication. National Science Foundation (1997). Available at: https://www.nsf.gov/pubs/1997/nsf97153/chap_4.htm (Accessed August 16, 2023).

[27] Angst J Azorin J-M Bowden CL Perugi G Vieta E Gamma A Prevalence and characteristics of undiagnosed bipolar disorders in patients with a major depressive episode the BRIDGE study. Arch Gen Psychiatry. (2011) 68(8):791–8. 10.1001/archgenpsychiatry.2011.87 21810644

[28] Daveney J Panagioti M Waheed W Esmail A. Unrecognized bipolar disorder in patients with depression managed in primary care: a systematic review and meta-analysis. Gen Hosp Psychiatry. (2019) 58:71–6. 10.1016/j.genhosppsych.2019.03.006 30933689

[29] Glick ID. Undiagnosed bipolar disorder: new syndromes and new treatments. Prim Care Companion J Clin Psychiatry. (2004) 6(1):27–33. 10.4088/PCC.v06n0106 15486598 PMC427610

[30] McIntyre RS Calabrese JR. Bipolar depression: the clinical characteristics and unmet needs of a complex disorder. Curr Med Res Opin. (2019) 35:1993–2005. 10.1080/03007995.2019.1636017 31311335

[31] Patel R Reiss P Shetty H Broadbent M Stewart R McGuire P Do antidepressants increase the risk of mania and bipolar disorder in people with depression? A retrospective electronic case register cohort study. BMJ Open. (2015) 5(12):e008341. 10.1136/bmjopen-2015 26667012 PMC4679886

[32] Alloy LB Ng TH Titone MK Boland EM. Circadian rhythm dysregulation in bipolar spectrum disorders. Curr Psychiatry Rep. (2017) 19(4):21. 10.1007/s11920-017-0772-z 28321642 PMC6661150

[33] Hirschfeld RMA Lewis L Vornik LA. Perceptions and impact of bipolar disorder: how far have we really come? Results of the national depressive and manic-depressive association 2000 survey of individuals with bipolar disorder. J Clin Psychiatry. (2003) 64(2):161–74. 10.4088/JCP.v64n0209 12633125

[34] Berk M Dodd S Callaly P Berk L Fitzgerald P de Castella AR History of illness prior to a diagnosis of bipolar disorder or schizoaffective disorder. J Affect Disord. (2007) 103:181–6. 10.1016/j.jad.2007.01.027 17324469

[35] Dagani J Signorini G Nielssen O Bani M Pastore A Girolamo GD Meta-analysis of the interval between the onset and management of bipolar disorder. Can J Psychiatry. (2017) 62:247–58. 10.1177/0706743716656607 27462036 PMC5407546

[36] Buoli M Cesana BM Fagiolini A Albert U Maina G de Bartolomeis A Which factors delay treatment in bipolar disorder? A nationwide study focussed on duration of untreated illness. Early Interv Psychiatry. (2021) 15(5):1136–45. 10.1111/eip.13051 33058435

[37] Stiles BM Fish AF Vandermause R Malik AM. The compelling and persistent problem of bipolar disorder disguised as Major depression disorder: an integrative review. J Am Psychiatr Nurses Assoc. (2018) 24:415–25. 10.1177/1078390318784360 29952230

[38] Price AL Marzani-Nissen GR. Bipolar disorders: a review (2012). Available at: www.aafp.org/afpAmericanFamilyPhysician483. (Accessed October 12, 2022). 22534227

[39] Rolin D Whelan J Montano CB. Is it depression or is it bipolar depression? J Am Assoc Nurse Pract. (2020) 32:703–13. 10.1097/JXX.0000000000000499 33017361

[40] Torres IJ Boudreau VG Yatham LN. Neuropsychological functioning in euthymic bipolar disorder: a meta-analysis. Acta Psychiatr Scand. (2007) 116:17–26. 10.1111/j.1600-0447.2007.01055.x 17688459

[41] Goldberg JF Ernst CL. What to do when your depressed patient develops mania. Fed Pract. (2016) 33(Suppl 2):26S–33S. PMID: 30766209; PMCID: PMC6375439 PMC6375439 30766209

[42] Mansell W Pedley R. The ascent into mania: a review of psychological processes associated with the development of manic symptoms. Clin Psychol Rev. (2008) 28:494–520. 10.1016/j.cpr.2007.07.010 17825463

[43] Andrade-González N Álvarez-Cadenas L Saiz-Ruiz J Lahera G. Initial and relapse prodromes in adult patients with episodes of bipolar disorder: a systematic review. Eur Psychiatry. (2020) 63(1):e12. 10.1192/j.eurpsy.2019.18 32093795 PMC7315869

[44] Poudyal A van Heerden A Hagaman A Islam C Thapa A Maharjan SM What does social support sound like? Challenges and opportunities for using passive episodic audio collection to assess the social environment. Front Public Health. (2021) 9:633606. 10.3389/fpubh.2021.633606 33855008 PMC8039317

[45] Wazen GLL Gregório ML Kemp AH Godoy MFd. Heart rate variability in patients with bipolar disorder: from mania to euthymia. J Psychiatr Res. (2018) 99:33–8. 10.1016/j.jpsychires.2018.01.008 29407285

[46] Richardson T Jansen M Fitch C. Financial difficulties in bipolar disorder part 1: longitudinal relationships with mental health. J Ment Health. (2018) 27(6):595–601. 10.1080/09638237.2018.1521920 30445874

[47] Richardson T Jansen M Fitch C. Financial difficulties in bipolar disorder part 2: psychological correlates and a proposed psychological model. J Ment Health. (2021) 30(1):3–11. 10.1080/09638237.2019.1581350 30955385

[48] Johnson SL Carver CS. Extreme goal setting and vulnerability to mania among undiagnosed young adults. Cognit Ther Res. (2006) 30:377–95. 10.1007/s10608-006-9044-7 20198117 PMC2829854

[49] Zhang J Pan Z Gui C Xue T Lin Y Zhu J Analysis on speech signal features of manic patients. J Psychiatr Res. (2018) 98:59–63. 10.1016/j.jpsychires.2017.12.012 29291581

[50] Low DM Bentley KH Ghosh SS. Automated assessment of psychiatric disorders using speech: a systematic review. Laryngoscope Investig Otolaryngol. (2020) 5:96–116. 10.1002/llio2.354 32128436 PMC7042657

[51] Weiner L Guidi A Doignon-Camus N Giersch A Bertschy G Vanello N. Vocal features obtained through automated methods in verbal fluency tasks can aid the identification of mixed episodes in bipolar disorder. Transl Psychiatry. (2021) 11(1):415. 10.1038/s41398-021-01535-z 34341338 PMC8329226

[52] Aan het Rot M Hogenelst K Schoevers RA. Mood disorders in everyday life: a systematic review of experience sampling and ecological momentary assessment studies. Clin Psychol Rev. (2012) 32(6):510–23. 10.1016/j.cpr.2012.05.007 22721999

[53] Dominiak M Kaczmarek-Majer K Antosik-Wójcińska AZ Opara KR Olwert A Radziszewska W Behavioral and self-reported data collected from smartphones for the assessment of depressive and manic symptoms in patients with bipolar disorder: prospective observational study. J Med Internet Res. (2022) 24(1):e28647. 10.2196/28647 34874015 PMC8811705

[54] Dunster GP Swendsen J Merikangas KR. Real-time mobile monitoring of bipolar disorder: a review of evidence and future directions. Neuropsychopharmacology. (2021) 46(1):197–208. 10.1038/s41386-020-00830-5 32919408 PMC7688933

[55] Roussos G Herrero TR Hill DL Dowling Av Müller MLTM Evers LJW Identifying and characterising sources of variability in digital outcome measures in Parkinson’s disease. NPJ Digit Med. (2022) 5(1):93. 10.1038/s41746-022-00643-4 35840653 PMC9284971

[56] Soreca I Frank E Kupfer DJ. The phenomenology of bipolar disorder: what drives the high rate of medical burden and determines long-term prognosis? Depress Anxiety. (2009) 26(1):73–82. 10.1002/da.20521 18828143 PMC3308337

[57] Levenson J Frank E. Sleep and circadian rhythm abnormalities in the pathophysiology of bipolar disorder. Curr Top Behav Neurosci. (2011) 5:247–62. 10.1007/7854_2010_50 25236559

[58] Matthews M Abdullah S Murnane E Voida S Choudhury T Gay G Development and evaluation of a smartphone-based measure of social rhythms for bipolar disorder. Assessment. (2016) 23(4):472–83. 10.1177/1073191116656794 27358214 PMC6155452

[59] Tanaka T Kokubo K Iwasa K Sawa K Yamada N Komori M. Intraday activity levels may better reflect the differences between major depressive disorder and bipolar disorder than average daily activity levels. Front Psychol. (2018) 9:2314. 10.3389/fpsyg.2018.02314 30581399 PMC6292921

[60] Dockendorf MF Hansen BJ Bateman KP Moyer M Shah JK Shipley LA. Digitally enabled, patient-centric clinical trials: shifting the drug development paradigm. Clin Transl Sci. (2021) 14(2):445–59. 10.1111/cts.12910 33048475 PMC7993267

[61] Barnett I Torous J Staples P Keshavan M Onnela JP. Beyond smartphones and sensors: choosing appropriate statistical methods for the analysis of longitudinal data. J Am Med Inform Assoc. (2018) 25(12):1669–74. 10.1093/jamia/ocy121 30272176 PMC6658863

[62] Nicholas J Boydell K Christensen H. Beyond symptom monitoring: consumer needs for bipolar disorder self-management using smartphones. Eur Psychiatry. (2017) 44:210–6. 10.1016/j.eurpsy.2017.05.023 28692910

[63] Stanislaus S Faurholt-Jepsen M Vinberg M Coello K Kjærstad HL Melbye S Mood instability in patients with newly diagnosed bipolar disorder, unaffected relatives, and healthy control individuals measured daily using smartphones. J Affect Disord. (2020) 271:336–44. 10.1016/j.jad.2020.03.049 32479333

[64] Burr C Morley J Taddeo M Floridi L. Digital psychiatry: risks and opportunities for public health and wellbeing. IEEE Trans Technol Soc. (2020) 1(1):21–33. 10.1109/tts.2020.2977059

[65] Morton E Nicholas J Lapadat L O’Brien HL Barnes SJ Poh C Use of smartphone apps in bipolar disorder: an international web-based survey of feature preferences and privacy concerns. J Affect Disord. (2021) 295:1102–9. 10.1016/j.jad.2021.08.132 34706421

[66] Garell C Svedberg P Nygren JM. A legal framework to support development and assessment of digital health services. JMIR Med Inform. (2016) 4(2):e17. 10.2196/medinform.5401 27226391 PMC4909384

