# Supplementary material for: Opportunities for digital health technology: identifying unmet needs for bipolar misdiagnosis and depression care management
Source: Front Digit Health. 2023 Sep 12;5:1221754. doi: 10.3389/fdgth.2023.1221754 (PMC10523347; doi:10.3389/fdgth.2023.1221754)
Supplement: Supplementary file 1 [file Datasheet1.pdf]

Supplementary Material

Opportunities for Digital Health Technology: Identifying Unmet Needs for Bipolar Misdiagnosis and Depression Care Management

Sarah M. Kark, Michelle A. Worthington, Richard H. Christie, Aaron J. Masino

Correspondence: Corresponding Author: sarahkark@gmail.com

1 Supplementary Appendix

MDD Psychiatrist Interview Guide

Clinician experience

- Can you take just a couple of minutes to tell me more about 1) your current practice and the patients you see, 2) a little bit about your training, and 3) your work in the area of MDD, particularly for outpatient MDD? What kind of patients do you typically see?
- How has your practice changed since COVID? Compared to sitting in the clinic with a patient and having them physically in front of you, what are the challenges, if any, of evaluating their clinical presentation remotely? Is there anything new you learn?
- How do you foresee telemedicine playing a permanent role in your practice?
- What do you and your patients need to enhance remote evaluation and treatment?
- Can you describe your typical care management process for an MDD patient? The kinds of patients you see, how you evaluate them, diagnosis, treatment plan, monitoring, and how you follow-up with them.
- What are the most important pieces of information you gather about a patient?
    - What are the key pieces of information for diagnosis?
    - How do you gather this information from your patients?
    - Can you tell me about how to differentiate between depressive symptoms and other underlying health problems, comorbid medical issues?
    - Can you tell me about how to differentiate between depressive symptoms and other psychiatric comorbidities?
- Can you please describe a few specific cases? What kinds of medications did you try with them over time and why? How long did it take to find a resolution?
- What kind of information do you use to choose a care management/treatment plan?
- What kind of information helps you feel confident in how you chose to treat a patient?
- What kind of information do you use to measure treatment outcomes? Can be quantitative and/or qualitative. Are there behaviors you observe that signal if they are improving?

- What kind of information do you use when you need to adjust a care plan?
- What kind of information helps you estimate how a patient will do on a new treatment?
- Can you tell me about patient follow-up? How do you monitor your patients and assess their response to treatment?
- What signs or behaviors–what do you listen and look for–that would prompt you to bring them back in early to change course?
- Once re-evaluated, how do you decide whether or not to change to a different medication or stay the course with the new medication?
- During the very early initial response phase, what is the most important information you use to predict how they might respond?
- If a patient does not respond to a new medication you try, what are the consequences and challenges associated with that?
- What are the consequences or problems of waiting 2-4 weeks to know if a new medication is working?
- Do you worry if they were remembering to take medications? What is working about this and what is not?
- Can you give me examples of information you wish you had about your patient in between clinic visits? Think with no bounds, the sky's the limit.
- What kind of information would you gather if you had more time to spend with each patient?
- Do you find your grouping patients into sub-categories? And how is that helpful to you? How does that guide how you assess or treat a patient?

Patient experience

- How would you describe care management / treatment experience from the patient point of view?
- Can you tell me about any feedback you have received from patients about the care management experience?

Hypothetical digital tools

Suppose your practice can prescribe an FDA-approved smartphone-based technology for remote patient monitoring. Using the app remotely, patients are asked to regularly complete tasks that gather video and audio data of their face, upper body, speech, and voice. Suppose the technology has the capability to use these data during the first 2 weeks of a new drug treatment to predict treatment response at 4 wks. Let's say you receive a notification 2 weeks into a new treatment that predicts non-response at 4 weeks:

- What would you do with this information?
- How would you act on it?
- If you knew the confidence of the non-response prediction for a specific instance, would that make any difference in your reaction?

- If you were also presented with the patient measurements that influenced the non-response prediction the most, would you find it helpful?
- Do you think it could help save time when deciding about the care management plan?
- Do you think it would be helpful to get examples of similar patient profiles and information about their treatment trajectory and outcome? How would you use this information?
- When would such a non-response warning be the most actionable for you? (e.g., # days after medication onset)?
- If a patient is not responding, would you worry if your patient is not taking the medication?
- What would you do with this non-response warning from the technology, if this was a new patient taking their first SSRI?
- What would you do with this non-response warning from the technology, if this was a patient with a history of polypharmacy?
- What else would you want to know about the factors that influenced the non-response prediction?
- How would that change your plan of action?

Building on the picture a bit more, let's say at 2 weeks you had observed some improvements in a patient taking the new drug, but a few days later you receive a non-response prediction warning from the technology:

- What would you do with this information?
- What else would you want to know about the factors that influenced the non-response prediction?
- How would that change your plan of action?
- When would such a non-response warning be the most actionable for you? (e.g., # days after medication onset)?
- Would you worry if your patient is not taking the medication?

Suppose a similar technology can use this video and audio data collected from patients to predict a risk probability score for a patient who is currently doing okay but shows signs that they will soon experience an increase in critical symptoms. The risk score is a warning of possible near-future increases in symptoms. Let's say that when the risk score goes above a certain threshold, your practice receives a notification that this patient might soon decline.

- What would you do with this risk score information?
- What if you had not observed a difference in their clinical presentation when you saw them recently, how would that influence your reaction?

Suppose using these remote video and audio measures gathered from the patient enables the technology to categorize your patients into specific subtypes of depression based on their data profile.

- How would you use this subtype information?
- What subtypes would be meaningful enough, if any, to inform your treatment selection with a patient?
- If you knew the confidence of the models' prediction for a specific instance, would that make any difference in your reaction?
- If the model presented to you the part of the patient measurements and history that has influenced the model decision the most, would you find it helpful?
- Do you think it could help save time when deciding on interventions?
- Do you think it would be helpful to get examples of similar patient profiles and information about their treatment trajectory and outcome? How would you use this information?

Building on this scenario a bit more, let's say your patient used the app for a week upon entering your practice, to collect a baseline. At your first appointment, the technology informs you that the patient falls into a particular subcategory of depression and displays the level of confidence.

  - How would you use this subtype information, perhaps in your evaluation or assessment or work-up?
  - How would this subtyping information inform your treatment selection?

Closing

- Is there anything we have not talked about that you would like to add?
- Is there anything else that you wish we knew?
